# Blood Screening for Influenza

**DOI:** 10.3201/eid1307.060861

**Published:** 2007-07

**Authors:** Michael Kai Hourfar, Anna Themann, Markus Eickmann, Pilaipan Puthavathana, Thomas Laue, Erhard Seifried, Michael Schmidt

**Affiliations:** * German Red Cross, Frankfurt, Germany; † Phillips University, Marburg, Germany; ‡ Mahidol University, Bangkok, Thailand; § QIAGEN, Hamburg, Germany

**Keywords:** avian influenza, influenza A (H5N1), blood screening, real-time PCR, dispatch

## Abstract

Influenza viruses, including highly pathogenic avian influenza virus (H5N1), could threaten blood safety. We analyzed 10,272 blood donor samples with a minipool nucleic acid amplication technique. Analytical sensitivity of the method was 804 geq/mL and 444 geq/mL for generic influenza primers and influenza (H5N1) subtype–specific primers. This study demonstrates that such screening for influenza viruses is feasible.

In the 20th century, 3 influenza-related pandemics occurred (1918 Spanish influenza, 1957 Asian influenza, and 1968 Hong Kong influenza) (*1*), which are now known to represent 3 different antigenic subtypes of the influenza A virus: H1N1, H2N2, and H3N2. Major influenza epidemics show neither periodicity nor a predictable pattern, and all differ from one another. Evidence suggests that true pandemics involving changes in hemagglutinin subtypes are caused by genetic reassortment in animal influenza A viruses. Since 2003, the World Health Organization has reported the infection of ≈218 persons and 124 deaths (56.9%; as of May 23, 2006) caused by the (H5N1) subtype in 10 different countries; a probable person-to-person transmission of the avian influenza virus was suggested (*2*). Most countries predicted death rates of 14–1,685 persons per 100,000 population in the event of a pandemic and estimated that up to 2,707 persons per 100,000 population would become infected (*3*).

Our study demonstrates that screening donor blood for influenza A (H5N1) subtype or for influenza viruses in general by minipool nucleic acid amplification technique (NAT) is feasible. To ensure the safety of blood products, this screening technique could be introduced into the blood-screening procedure without delay in the case of a pandemic.

## The Study

To increase blood safety, we introduced minipool NAT screening in our blood donor service in 1997 for hepatitis B virus (HBV), hepatitis C virus (HCV), and HIV-1 and in 2000 for hepatitis A virus (HAV) and parvovirus B19 (*4*). For these purposes, 100-μL aliquots of up to 96 blood samples were pooled. The complete pool of up to 9.6 mL was centrifuged at 58,000× *g* for 60 min at 4°C. Viruses were extracted by using spin columns, and nucleic acid was eluted in a total volume of 75 μL. Only 60 μL of extract is needed for routine NAT screening. A residual volume of 15 μL can then be used for additional NAT testing (*5*) for influenza viruses.

The real-time quantitative amplification of influenza/H5 was performed according to the manufacturer’s instructions (Artus Influenza/H5 LC RT-PCR Kit, QIAGEN, Hamburg, Germany) by using a thermocycler (LightCycler; Roche Applied Science, Mannheim, Germany). The test consists of 2 individual amplification reactions. In the first step, a generic influenza PCR is performed. The specificity of this reaction was demonstrated for all subtypes of influenza A (H1–H15, N1–N9) and all subtypes of influenza B. Samples with a positive test result in the first PCR were analyzed in a second PCR with influenza (H5N1)–specific primers and probes. Therefore, the assay allows differentiation between avian influenza (H5N1) and other influenza virus strains.

To mimic a situation like an H5-positive donation, a purified culture supernatant of Vero cells infected with influenza (H5N1) (strain A/Thailand/1 (KAN-1)/2004) (*6*) was used as an external quantification standard. Virion integrity in this preparation was confirmed by electron microscopy. The viral RNA concentration was determined in an external laboratory by multiple quantitative real-time PCR determinations (*7*). Different dilutions of the external influenza (H5N1) subtype quantification standard (0.0, 0.91, 1.96, 3.91, 7.81, 15.63, 31.25, 62.5, 125, and 250 PFU/mL) were prepared, and 100 μL of each dilution was spiked into 9.5-mL negative plasma pools. Each dilution was repeatedly spiked and tested in 8 minipools. Five microliters of the extract was analyzed with the generic influenza NAT as well as with the specific influenza (H5N1) NAT. Results are shown in Tables 1 and 2. Probit analysis of these data yielded a detection probability of >95% in parallel tests when an average of at least 13.4 PFU/mL (95% confidence interval [CI] 8.3–184 PFU/mL) and 7.4 PFU/mL (95% CI 5.2–14.7 PFU/mL) for influenza generic assay and for the influenza (H5N1)–specific test, respectively, were present in individual plasma samples before pooling.

**Table 1:** Analytical sensitivity for influenza virus in plasma samples*

PFU (H5N1) spiked in minipools	No. positive/ no. tested	% Positive
250.0	8/8	100
125.0	8/8	100
62.5	8/8	100
31.3	8/8	100
15.6	7/8	87.5
7.8	8/8	100
3.9	4/8	50
0.0	0/8	0

*Influenza (H5N1) standard was extracted from 9.6 mL of 96 pooled donor samples after centrifugation. Five microliters of 75-µL nucleic acid extract was analyzed. The 95% detection limit was 13.4 PFU/mL; the 50% detection limit was 4.8 PFU/mL.

**Table 2:** Analytical sensitivity for avian influenza (H5N1) virus subtype for plasma samples*

PFU (H5N1) spiked in minipools	No. positive/ no. tested	% Positive
125.0	8/8	100
62.5	8/8	100
31.3	8/8	100
15.6	8/8	100
7.8	7/8	87.5
3.9	7/8	87.5
1.9	4/8	50
0.9	3/8	37.5
0.0	0/8	0

*Influenza (H5N1) standard was extracted from 9.6 mL of 96 pooled donor samples after centrifugation. Five microliters of 75-µL nucleic acid extract was analyzed. The 95% detection limit was 7.4 PFU/mL; the 50% detection limit was 2.5 PFU/mL.

A total of 117 routine minipools, representing 10,272 blood donor samples, containing an average of 88 ± 8 samples per pool, had previously been tested for HIV-1, HBV, HCV, HAV, and parvovirus B19. All pools were negative for influenza virus when tested with the generic influenza PCR and the influenza (H5N1)–specific PCR. One pool had invalid results (failed amplification of internal control RNA, representing 0.01% of all analyzed runs).

## Conclusions

As reported by AuBuchon et al. (*8*), NAT significantly increased the safety of blood products. At the German Red Cross, look-back examinations showed only 1 transfusion had transmitted HIV-1 (1998) after the introduction of NAT testing. Blood donor screening by NAT was made technically and financially feasible by creating minipools of up to 96 individual samples per pool. Roth et al. demonstrated an efficient enrichment for all tested viruses in plasma samples (*9*). In the absence of an infective donor, different concentrations of the new influenza genotype H5N1 were spiked into minipools of 95 samples. As shown in Table 2, the influenza (H5N1) subtype was detected by the generic influenza primers as well as by the influenza (H5N1)–specific primers when our routine minipool screening procedure was used. Sensitivity was expressed as PFU/mL and can be converted into viral genome copy number according the calculation of Yoshikawa et al. (*7*). Therefore, the analytical sensitivity was ≈804 geq/mL and 444 geq/mL for a generic influenza and for the influenza (H5N1) subtype, respectively.

After screening 10,272 samples by minipool NAT, none of the samples were found to be infected by influenza, which corresponds with the low EISS Index (European Influenza Surveillance Scheme index) of <20 during the study period (February–April, 2006) (*10*). An EISS index >80 is expected during an influenza epidemic, as was seen in 2005. Therefore, blood screening should be repeated during the next acute influenza season.

Accepted incubation periods for influenza range from 2 to 10 days (*11*,*12*). As with other viruses, a viremic phase of infection can be assumed to precede clinical symptoms such as fever (*13*,*14*). Recently Chutinimitkul et al. (*15*) detected influenza (H5N1) virus (3,080 copies/mL) in the plasma of a 5-year-old boy, which indicates a viremic phase of influenza (H5N1) infection. Those donors may be infective, especially to immunosuppressed patients. In addition to quarantine of infected patients, treatment with antiviral drugs, and development of avian influenza vaccines, blood donors should be tested during a pandemic to avoid transfusion-transmitted infections. Our study demonstrates that NAT screening could be incorporated into blood testing without delay and that the influenza virus could be sufficiently enriched by centrifugation. Sensitivity of our influenza-screening method would have been sufficient to detect recently reported virus concentrations in plasma of infected persons (*15*). However, as with all minipool methods, infections can be transmitted to transfusion recipients on rare occasions because the viremia level in the donor is below the analytical sensitivity of the screening assay.

To reduce this risk, a selective infectious dose NAT strategy (e.g., triggering of infectious dose NAT testing when at least 1 viremic donation is collected per week with the standard minipool screening algorithm), as performed for West Nile virus (WNV) screening in the United States might be necessary. Implementation of WNV-NAT in the United States in 2003 interdicted well over 1,000 donations from persons infected with WNV and is a good example of successful implementation of NAT screening for emerging viruses.

The collective fight against new viruses such as severe acute respiratory syndrome virus, WNV, or influenza (H5N1) presents an immense challenge for the whole community, but new molecular-biologic methods offer opportunities to overcome this challenge. NAT screening tests are now available soon after the sequencing of new viruses. In the absence of a general pathogen inactivation method for all blood products (erythrocytes, platelets, and plasma), the NAT screening procedure allows testing for new viruses to ensure blood safety.
